# Dietary Supplements in Cardiovascular and Metabolic Diseases

**DOI:** 10.3390/nu16101418

**Published:** 2024-05-08

**Authors:** Bruno Trimarco, Gaetano Santulli

**Affiliations:** 1 Department of Advanced Biomedical Sciences, Federico II University Hospital, 80131 Naples, Italy; bruno.trimarco@unina.it; 2 International Translational Research and Medical Education (ITME) Consortium, Academic Research Unit, 80100 Naples, Italy; 3 Department of Medicine, Fleischer Institute for Diabetes and Metabolism (FIDAM), Albert Einstein College of Medicine, New York, NY 10461, USA; 4 Department of Molecular Pharmacology, Wilf Family Cardiovascular Research Institute, Einstein Institute for Aging Research, Einstein-Mount Sinai Diabetes Research Center (ES-DRC), Einstein Institute for Neuroimmunology and Inflammation (INI), Albert Einstein College of Medicine, New York, NY 10461, USA

Recent research has sparked increasing interest in the effects of dietary supplements on cardiovascular and metabolic disorders. The aim of this Special Issue was to compile top-tier research papers with a strong foundation in basic research and/or potential for translation in this area, to offer an up-to-date systematic examination of the functional role of dietary supplements in cardiovascular and metabolic disease. We included both clinical and pre-clinical studies on this compelling topic.

The Special Issue begins with two preclinical investigations studying the effects of the extract of the bergamot fruit (*Citrus bergamia*) in vitro [1] and in vivo [2], observing a reduction in oxidative stress (research led by Dr. Speranza Rubattu [1]) and dyslipidemia (research led by Vincenzo Mollace [2]). Dr. Mollace’s group also contributed a review on the potential therapeutic approach of combining SGLT2 inhibitors and plant extracts [3].

In another in vivo study, led by Dr. Francisco Javier Pavón-Morón, humanized mice were supplemented with L-carnitine and orally administered essential oil emulsions derived from patients with ischemic heart disease and type 2 diabetes mellitus for 40 days [4]. The researchers evaluated the impact on the gut microbiota composition, microbial metabolites, and plasma markers related to cardiovascular disease, inflammation, and oxidative stress. The results indicated that essential oil emulsions, particularly those containing parsley and rosemary essential oils, exhibited prebiotic effects on beneficial commensal bacteria, primarily of the Lactobacillus genus; moreover, mice treated with these essential oils showed a decrease in plasma trimethylamine N-oxide (TMAO) levels and an increase in fecal short-chain fatty acids (SCFAs) levels [4]. An assay in Wistar–Kyoto rats demonstrated that increased oxidative stress accompanied the intake of copper nanoparticles, which further modulated vascular relaxation with the participation of 20-hydroxyeicosatetraenoic acid (20-HETE), through the thromboxane-A_2_ receptors [5]. Another study evaluated the quality of 19 products, including herbal teas and dietary supplements, by assessing the content of 1-deoxynojirimycin (DNJ, known for its ability to inhibit α-glucosidase and regulate postprandial glucose levels), selected (poly)phenols, and antioxidant activity [6]. The results revealed subpar quality in many dietary supplements, potentially compromising their health benefits, due to the low nutraceutical content. Furthermore, a novel method utilizing ATR-FTIR spectroscopy combined with PLS regression for DNJ content determination was proposed, offering a rapid screening tool for assessing product quality without the need for complex chromatographic processes.

Two reviews are included in the Special Issue. The first one considered astaxanthin, a natural carotenoid commonly found in various aquatic animals, including salmon, shrimp, and crustaceans, which shows a very strong antioxidant effect that is 14, 65, and 54 times higher than that of vitamin E, C, and β-carotene, respectively [7]. The second one explored the role of taurine in aging and cardiovascular health [8].

Next, the Special Issue presents clinical studies. The first study was a retrospective analysis of 685,778 patients newly diagnosed with osteoarthritis, showing that the adherent usage of glucosamine was significantly associated with a higher risk for cardiovascular diseases in patients with osteoarthritis [9]. A single-blind randomized controlled trial, coordinated by Dr. Francesco Landi, demonstrated that L-Arginine plus vitamin C supplementation (an association that has been shown to be effective in long-COVID [10,11,12,13] by attenuating endothelial dysfunction and oxidative stress [13,14,15,16]) can improve walking performance, muscle strength, and fatigue in adults with long-COVID [17].

The Special Issue concludes with the presentation of three randomized, double-blind, and placebo-controlled trials. The first trial tested the effects of dietary Coenzyme Q10 plus NADH supplementation on the fatigue perception and health-related quality of life in patients with myalgic encephalomyelitis/chronic fatigue syndrome [18]. A 12-week clinical trial was conducted with 207 participants, showing significant reductions in cognitive fatigue perception and overall fatigue severity, as well as improvements in the health-related quality of life among those receiving CoQ10 and NADH; additionally, improvements in sleep duration and efficiency were observed in the experimental group [18]. 

The second trial aimed to assess the impact of water-soluble tomato concentrate (WSTC) from fresh tomatoes on platelet apoptosis and oxidative stress in a clinical trial lasting 10 weeks and involving 52 healthy middle-aged and elderly adults, revealing that WSTC supplementation for four weeks significantly increased the serum total antioxidant capacity levels and reduced the serum malondialdehyde levels [19]. 

The third trial demonstrated that dietary supplementation with standardized bergamot polyphenolic fraction phytosome, artichoke extracts, Q10 phytosome, and zinc safely exerted significant improvements in serum lipids, systemic inflammation, indexes of non-alcoholic fatty liver disease (NAFLD), and endothelial reactivity in healthy subjects with moderate hypercholesterolemia [20].
